# Experimental infection of horses with *Rickettsia rickettsii*

**DOI:** 10.1186/s13071-016-1784-y

**Published:** 2016-09-13

**Authors:** Tatiana Evelyn Hayama Ueno, Francisco B. Costa, Jonas Moraes-Filho, Washington Carlos Agostinho, Wilson Roberto Fernandes, Marcelo B. Labruna

**Affiliations:** 1 Agribusiness Technology Agency of São Paulo State, São José do Rio Preto, Brazil; 2 Faculty of Veterinary Medicine, University of São Paulo, São Paulo, Brazil

**Keywords:** *Rickettsia rickettsii*, Rocky mountain spotted fever, Horses, Ticks, *Amblyomma cajennense*, Brazil

## Abstract

**Background:**

*Rickettsia rickettsii* is vectored by ticks, and some vertebrate hosts can be sources of infection to ticks during bacteremic periods. In Brazil, the main vector for *R. rickettsii* is the tick *Amblyomma sculptum*, a member of the *A. cajennense* complex. Horses, in turn, are one of the major hosts for *A. sculptum*. In this study, horses experimentally infected with *R. rickettsii* were assessed for clinical changes and their capability to transmit the infection to *A. sculptum* ticks.

**Methods:**

Four horses were infected with *R. rickettsii* through either intraperitoneal injection or infestation with *R. rickettsii-*infected *A. sculptum* ticks. Simultaneously, the animals were infested with non-infected *A. sculptum* ticks. The horses were monitored for 30 days by clinical examination, hematological and biochemical tests, real-time PCR of blood for the detection of *Rickettsia*, and inoculation of blood in guinea pigs. IgG antibody titers were followed until the horses have shown seronegativity or until the end of the experiment. Uninfected ticks that fed on horses were subjected to real-time PCR and/or were fed on susceptible rabbits.

**Results:**

The horses showed no clinical, hematological or blood biochemical alterations, and bacteremia was not detected by real-time PCR or by inoculation of horse blood into guinea pigs. Anti-*R. rickettsii* antibodies were detected in horses from 10 days to 2 years after infection. Uninfected ticks, after feeding on infected horses, showed 2.1 % positivity in real-time PCR, but failed to transmit the infection to rabbits at a next feeding stage.

**Conclusions:**

*Rickettsia rickettsii*-infected horses did not manifest illness and are not competent amplifier hosts of *R. rickettsii* for *A. sculptum* ticks.

## Background

The bacterium *Rickettsia rickettsii* is the etiological agent of a severe illness in humans, known as Brazilian spotted fever (BSF) in Brazil or Rocky Mountain spotted fever in the USA [1]. In Brazil, the disease was first reported during the 1920s in the state of São Paulo [2], the region with the largest number of notifications throughout the history of this country [3].

In South America, the main vectors of *R. rickettsii* are ticks of the *Amblyomma cajennense* species complex, also called as *A. cajennense* (*sensu lato*) (*s.l*.) [1, 4, 5]. This species complex occurs only in the Americas and is distributed from southern USA to northern Argentina [4, 5]. Recently, the *A. cajennense* species complex was split into six species: *A. cajennense* (*sensu stricto*) (*s.s*.), *A. sculptum*, *A. interandinum*, *A. mixtum*, *A. patinoi* and *A. tonelliae* [5]. In the state of São Paulo, southeastern Brazil, this species complex is represented only by *A. sculptum,* which is therefore, the main vector of *R. rickettsii* in this part of South America [5, 6]. Hence, all previous studies with *A. cajennense* (*s.l*.) in southeastern Brazil were in fact related to *A. sculptum* [6].

Ticks are natural reservoirs of *R. rickettsii* and may remain infected for life. They acquire the infection by transovarial transmission or through feeding on bacteremic vertebrate animals, which are called amplifier hosts [1, 7]. In the case of *A. sculptum* (reported as *A. cajennense*), the second mechanism is essential for the maintenance of the bacterium in the environment because transovarial transmission rate is low and infected ticks have reduced fertility [8]. In the USA, experimental infections showed that several small rodent species were able to transmit the bacterium to *Dermacentor andersoni* or *D. variabilis* ticks [9–11]. In Brazil, capybaras (*Hydrochoerus hydrochaeris*) and opossums (*Didelphis aurita*) were confirmed as amplifying hosts for *A. sculptum* ticks (published as *A. cajennense*) [12, 13].

Horses are one of the main hosts of all parasitic stages of *A. sculptum* (reported as *A. cajennense*) in many BSF-endemic regions [14], where high frequencies of *R. rickettsii*-seropositive horses are usually found [15–17]. In previous studies in North America, *R. rickettsii-*experimentally infected horses manifested fever and had rickettsemia for only one day; ticks, however, were not used in these earlier studies [18, 19].

We aimed to investigate the clinical findings in horses experimentally infected with *R. rickettsii* and the role of horses as amplifier hosts of the bacterium for *A. sculptum* ticks.

## Methods

### Animals

Four adult crossbred horses were used. Paired serum samples from each animal, taken 15 days apart, proved to be negative through indirect immunofluorescence assay (IFA) [16] (protocol described below) against antigens from six *Rickettsia* species occurring in Brazil: *R. rickettsii*, *R. parkeri*, *R. amblyommii*, *R. rhipicephali*, *R. felis* and *R. bellii*. During the first 30 days of the experiment (period with artificial tick infestations), horses were housed individually in an isolation stall. After 30 days (at the end of infestations), horses were sprayed with acaricide and transported to a pasture, where they were held until the end of the experiment. Guinea pigs and New Zealand white rabbits were purchased from commercial breeding centre and were housed individually.

#### *Rickettsia rickettsii*

The strain Taiaçu of *R. rickettsii*, originally isolated from the tick *A. aureolatum* in Brazil, was used in this study [20]. This strain has been maintained by consecutive passages in guinea pigs [8] or Vero cells [21]. Because the lineage maintained in guinea pigs probably better preserves the virulence of bacterium, it was used in experimental infections. The Vero cell lineage was used as antigen in IFA.

To prepare the inoculum, guinea pigs previously infected with *R. rickettsii* were euthanized on the third day of fever and then aliquots of spleen, liver, lung and brain were collected and stored at -80 °C. At the moment of use, the organs were thawed, pooled, macerated with brain heart infusion (BHI), and immediately inoculated in the horses or guinea pigs [8], as described below.

#### *Amblyomma sculptum* ticks

*Amblyomma sculptum* adult ticks were collected by dragging in Pedreira municipality, a BSF-endemic area of São Paulo, and used to form a laboratory colony. The ticks were identified according to taxonomic key [22] and a recent description/redescription of members of the *A. cajennense* species complex [5]. Uninfected ticks were sequentially fed on rabbits and maintained in an incubator at 25 °C and a relative humidity of 85 ± 5 %, until obtaining unfed larvae, nymphs and adults. To confirm that the colony was not naturally infected, rabbits used for feeding ticks were tested for antibodies against *R. rickettsii*, *R. parkeri*, *R. amblyommii*, *R. rhipicephali*, *R. felis* and *R. bellii* [16] before and 21 days after infestation, and showed to be negative. Furthermore, rickettsial DNA was not detected by real-time PCR [21] (protocol described below) in female ticks after oviposition.

To produce *R. rickettsii-*infected ticks, part of uninfected larvae were fed on guinea pigs inoculated with *R. rickettsii*. The engorged larvae were collected daily. After molting to nymphs in an incubator, a sample of five ticks corresponding to each detachment day from guinea pigs (total of 60 tested nymphs) was subjected to real-time PCR [21] to verify the infection success. The proportion of positive nymphs was 8.3 %, ranging from 0 to 40 % according to detachment day. Only ticks that were collected at days 4 and 5 post-infestation showed positive samples. The nymphs corresponding to these days were fed on new inoculated guinea pigs. The engorged nymphs were collected daily and after molting to adults, a sample of four to eight ticks corresponding to each detachment day from guinea pigs was tested by real-time PCR [21], resulting in 49 tested adult ticks. The infection rate of these adult ticks by real-time PCR was 89.8 % (44/49), ranging from 20 through 100 %, according to detachment day. Ticks corresponding to the days with 100 % positive samples were selected for experimental infestations of horses, as described below.

### Experimental groups

The four horses were divided into two groups. In Group 1 (horses 1 and 2), two cotton sleeves for tick infestation were glued to the shaved skin of each horse in the paravertebral region. On day 0, 30 males and 30 females of the *A. scupltum-*infected colony were placed into one of the sleeves. Male ticks were allowed to feed until detachment of the last engorgement female, 24 days after infestation. In the second sleeve, approximately 1,000 larvae, 200 nymphs and 10 adult couples of non-infected ticks were placed on day 2. This infestation was repeated in the same sleeve on days 7, 12, 17 and 22.

In Group 2 (horses 3 and 4), only one infestation sleeve was attached to each horse. On day 0 each horse was inoculated intraperitoneally with a homogenate of *R. rickettsii-*infected guinea pig organs, as detailed previously [8]. On the same day, approximately 1,000 larvae, 200 nymphs and 10 adult couples of non-infected ticks were placed into the sleeve. This infestation was repeated on days 5, 10, 15 and 20.

All horses were clinically examined and their rectal temperatures were measured once a day [23], from 0 through 30 days post-inoculation or post-infestation (dpi). Blood samples were collected from the jugular vein of all horses, every 2 days, from 0 through 30 dpi. Aliquots of blood were distributed as follows: whole blood with ethylenediaminetetraacetic acid (EDTA) for hematological analysis and detection of *Rickettsia* by real-time PCR [21]; heparinized whole blood for inoculation in guinea pigs; and serum for the detection of anti-*R. rickettsii* antibodies by IFA [16]. Serum biochemical tests were performed on days 0, 6, 12, 18, 24 and 30. After 30 days of experiment, the blood continued to be collected for monitoring the antibody curve every 7 days until 128 dpi, and then at intervals of 7–42 days until the animals became seronegative or the work was ended.

Sera from five time-points were selected to be tested simultaneously against *R. rickettsii*, *R. parkeri*, *R. amblyommii*, *R. rhipicephali*, *R. bellii* and *R. felis* [16], in order to compare endpoint titers: (i) day 0; (ii) the first day of animal seropositivity to *R. rickettsii*; (iii) the day when titers peaked to *R. rickettsii*; (iv) someday in the decreasing phase of titer; and (v) the last day of seropositivity or animal monitoring.

### Hematological and biochemical analyses

Hematological analysis was performed manually as previously described [24]. Commercial kits were used in the biochemistry tests to quantify serum concentrations of urea, creatinine (DiaSys, Holzheim, Germany), aspartate aminotransferase, total and conjugated bilirubin, total protein, albumin (BioSystems, Barcelona, Spain), and gamma-glutamyl transferase (Randox, Crumlin, Northern Ireland); the readings were done in a LabMax 240 automatic analyzer (Labtest, Lagoa Santa, Brazil). Results were compared with horse reference values [25, 26].

### Inoculation of horse blood into guinea pigs

Two guinea pigs were each inoculated intraperitoneally with 500 μl of horse blood after each collection. Guinea pigs were clinically monitored and their rectal temperatures were measured up to 21 dpi, when they were tested for antibodies against *R. rickettsii* [16]. The animal was considered febrile if temperature exceeded 40 °C [27].

### Evaluation of tick acquisition and transmission of rickettsiae

In the two horse groups, the sleeve that received uninfected ticks was opened daily for recovering engorged larvae, nymphs and females. Males and non-engorged females that were still attached were removed from horses after 30 dpi and were tested in conjunction to engorged females after oviposition by real-time PCR [21]. Nymphs and adults that molted from engorged larvae and nymphs, respectively, were fed on rabbits. Two rabbits were used for ticks of each horse, the first one receiving ticks that detached from horse up to 15 dpi, and the second rabbit receiving ticks that detached from 16 to 30 dpi. The rabbits had their rectal temperature measured for 21 days and their serum samples were subjected to IFA [16] to detect anti-*R. rickettsii* antibodies at 0 and 21 dpi. Rabbits were considered febrile if temperature exceeded 40 °C [27]. After feeding on rabbits, male ticks and females that did not become fully engorged were manually removed and tested by real-time PCR [21]. Engorged nymphs and females recovered from rabbits were also tested by real-time PCR [21] after molting to adults or completing oviposition, respectively.

Only for horse 1, a sample of 90 unfed nymphs and 56 unfed adults, resulting from molting of engorged larvae and nymphs, respectively, was subjected to real-time PCR [21]. The remaining unfed ticks were fed on the rabbits. For the other three horses, all unfed nymphs and adults were placed on rabbits because fewer engorged ticks were recovered from these horses.

**Fig. 1:**
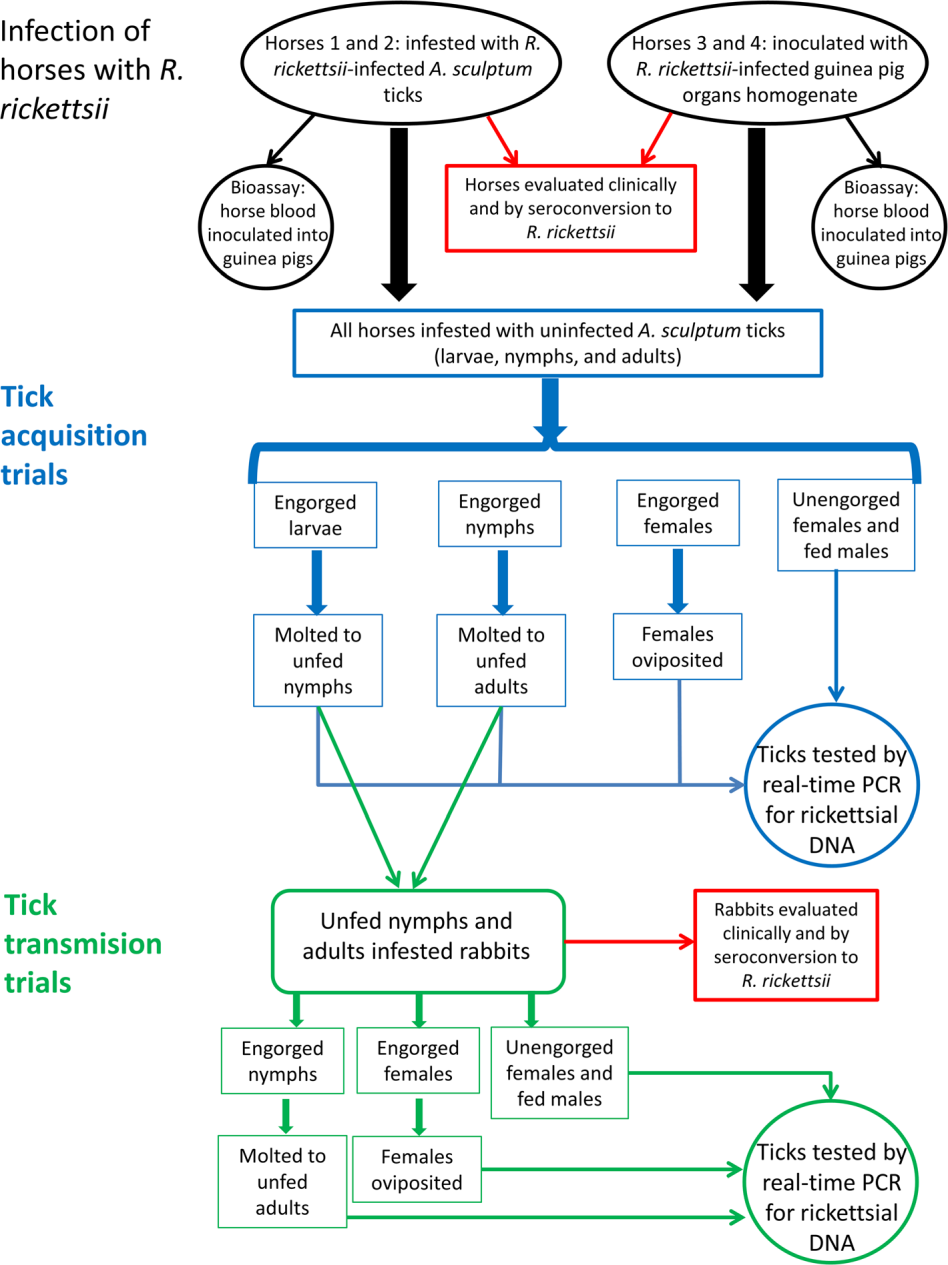
Diagram illustrating experimental procedures of the present study. Two horses (Group 1) were exposed to rickettsial infection through infestation with *Rickettsia rickettsii-*infected *Amblyomma sculptum* ticks. Two other horses (Group 2) were infected through intraperitoneal inoculation of a homogenate of *R. rickettsii-*infected guinea pig organs. The four horses were clinically evaluated and infested with uninfected *A. sculptum* ticks (larvae, nymphs and adults) during 30 days. Recovered ticks were reared to the next developmental stage and/or tested by real-time PCR for detection of rickettsial DNA. Molted, unfed ticks were allowed to feed on tick-naïve rabbits, which were clinically evaluated for 21 days and tested by seroconversion through testing paired serum samples (days 0 and 21 post-infestation) against *R. rickettsii* antigens. Solely for horse 1, a sample of unfed nymphs and adults, resulting from molting of engorged larvae and nymphs, respectively, was subjected to real-time PCR, and the remaining unfed ticks were fed on the rabbits. For the other three horses, all unfed nymphs and adults were placed on rabbits

Positive ticks in real-time PCR [21] were subjected to conventional PCR [28, 29] and sequencing of the *Rickettsia ompA* gene, as described below [28, 29]. Procedures with ticks, horses and rabbits are summarized in Fig. 1.

### Infectivity test of ticks and inocula

To confirm the viability of rickettsiae in the colony of infected ticks, for each horse in the Group 1 (inoculated via infected ticks), one rabbit was infested simultaneously with five tick couples from this colony. The rabbits were examined daily and their serum samples were subjected to IFA [16] for *R. rickettsii* before and after infestation. To verify whether the ticks were indeed infected, male ticks and females that did not become fully engorged were manually removed from rabbits and horses, and tested by real-time PCR [21]; fully engorged females were tested at the end of oviposition. Among the infected adult ticks that fed on horses, six (three from each horse) were selected for isolation of the bacterium in cell cultures [21, 30] and guinea pig inoculation [20]. Isolations were confirmed by sequencing of the rickettsial *ompA* gene [28, 29] from cell cultures and spleen or liver of guinea pigs.

For each horse from Group 2 that was intraperitoneally inoculated with *R. rickettsii*, guinea pigs (three guinea pigs for horse 3 and two guinea pigs for horse 4) were simultaneously inoculated with an aliquot of the same homogenate of *R. rickettsii*-infected guinea pig organs that was inoculated into horses, to verify the inoculum viability. Guinea pigs were clinically examined until the 21st dpi, when they were tested for anti-*R. rickettsii* antibodies by IFA [16].

### IFA

IFA tests were performed using crude antigens of *R. rickettsii*, *R. parkeri*, *R. amblyommii*, *R. rhipicephali*, *R. bellii* and *R. felis* [16]. As secondary antibody, commercial anti-horse IgG, anti-rabbit IgG, or anti-guinea pig IgG antibody (Sigma-Aldrich, St. Louis, MO, USA) was used. Serum samples were initially tested at the 1:64 dilution. Positive samples at initial screening were serially diluted for the determination of endpoint titers. Positive and negative control sera corresponding to each animal species were added to each IFA slide.

### PCR

DNA extraction from horse blood and guinea pig organs was performed with the commercial kit DNeasy Blood & Tissue Kit (QIAGEN, Hilden, Germany), according to the manufacturer’s instructions. The DNA of ticks (processed individually) and cell cultures were extracted by using guanidine isothiocyanate-phenol solution [31]. For detection of the *Rickettsia* spp. in blood, ticks, cell culture and organs, we used a TaqMan real-time PCR assay targeting a 147 bp fragment of the *gltA* gene [21]. Each reaction was prepared in a final volume of 25 μl, containing 2.5 μl of 10× buffer, 2 mM of MgCl_2_, 0.2 mM of each deoxynucleotide triphosphate (dNTP), 0.6 μM of each primer, 0.1 μM of probe, 0.03 U/μl of Platinum *Taq* DNA polymerase (Invitrogen, Carlsbad, CA, USA), 2.5 μl of DNA template, and 11.65 μl of water. Reactions were performed in the 7500 Real Time PCR System apparatus (Applied Biosystems, Foster City, CA, USA) under the following conditions: 1 cycle at 50 °C for 2 min and 95 °C for 10 min, followed by 40 cycles of 95 °C for 15 s and 60 °C for 1 min. In each run, one positive control (DNA of *R. parkeri* strain NOD) and three negative controls (water) were added. Positive samples in real-time PCR were subjected to conventional PCR and sequencing [21] of a fragment of the rickettsial *ompA* gene for comparison with GenBank sequences and confirmation of the *Rickettsia* species. The reaction mixture of *ompA*-based PCR was prepared in a final volume of 25 μl, containing 2.5 μl of 10× buffer, 2 mM MgCl_2_, 0.25 mM of each dNTP, 0.6 μM of primers 190.70 [28] and 190.701 [29], 0.03 U/μl of Platinum *Taq* DNA polymerase, 2.5 μl of DNA template, and 10.85 μl of water. The following conditions were used in the Mastercycler Gradient thermocycler (Eppendorf, Hamburg, Germany): 1 cycle at 95 °C for 5 min, followed by 35 cycles at 95 °C for 40 s, 58 °C for 30 s, and 65 °C for 45 s, and a final extension at 72 °C for 10 min.

Ticks negative in real-time PCR were subjected to conventional PCR targeting mitochondrial *16S* rDNA of ticks [32]. The reaction mixture contained the same reagent concentrations used for *ompA* PCR. The reactions were performed under the following conditions: 1 cycle at 94 °C for 3 min, 10 cycles of 94 °C for 30 s, 98 °C for 30 s, and 72 °C for 40 s, 15 cycles of 94 °C for 30 s, 50 °C for 30 s, and 72 °C for 40 s, 10 cycles of 94 °C for 30 s, 55 °C for 30 s, and 72 °C for 40 s, and finally 1 cycle at 72 °C for 7 min. If the sample was positive, the negative result for the *Rickettsia-*real-time PCR assay was validated. If the sample was negative, the DNA extraction was considered ineffective and the sample was discarded from the study [8].

## Results

### Horse clinical aspects

**Fig. 2:**
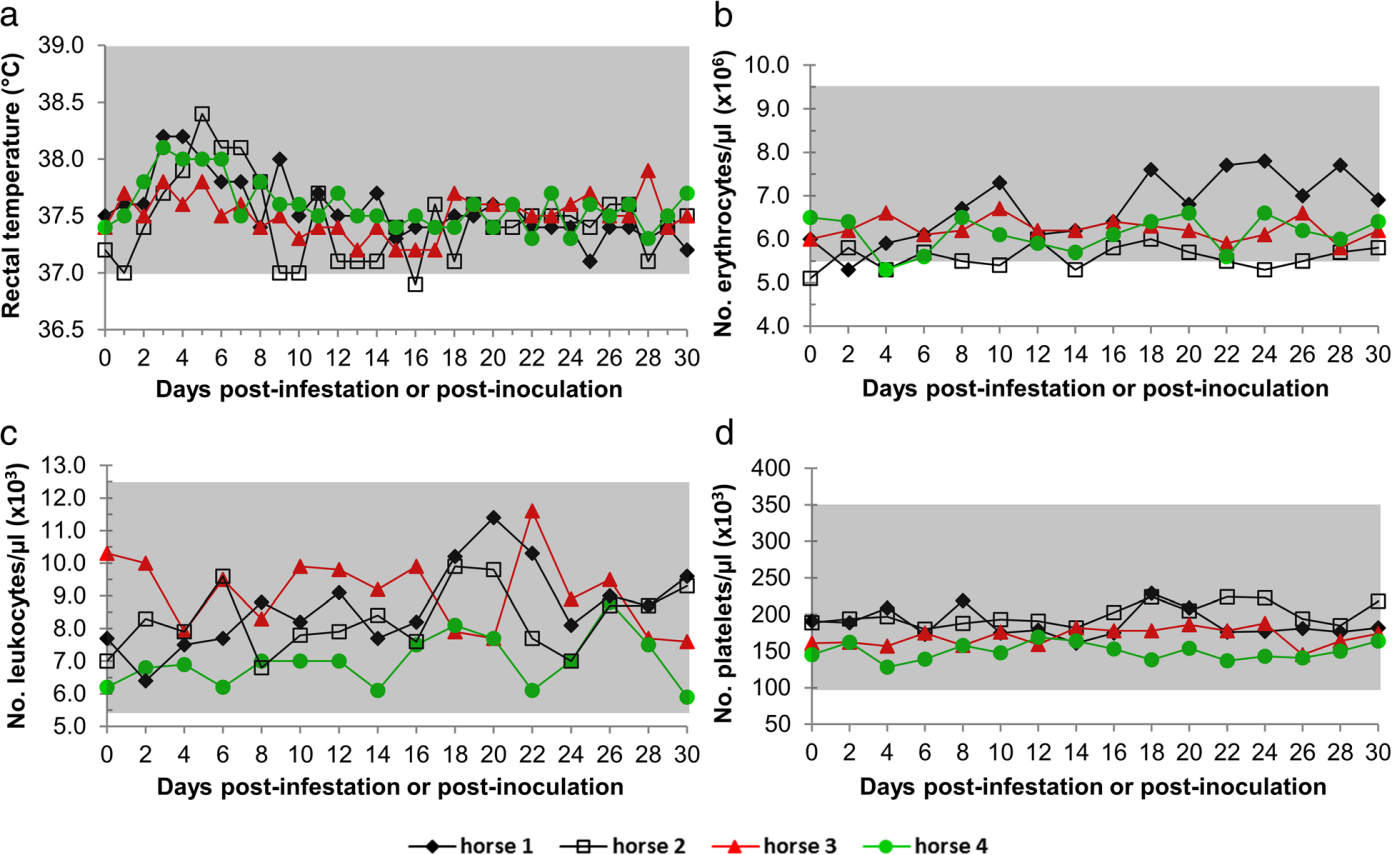
Rectal temperature (**a**) and hematological values (**b**-**d**) in four horses experimentally infected with *Rickettsia rickettsii*, during 30 days of monitoring. Horses 1 and 2 (Group 1) were infested with *R. rickettsii*-infected *Amblyomma sculptum* ticks. Horses 3 and 4 (Group 2) were inoculated intraperitoneally with macerated organs from *R. rickettsii*-infected guinea pigs. Gray areas represent the reference ranges for horses [23, 24, 40]

All four horses did not show consistent clinical changes. Some clinical and laboratory measurements were slightly different from reference values, but without clinical relevance, as they were observed before and after inoculation or infestation or on sparse days. Rectal temperature values were never above the upper reference limit, although there was a slight increase (temperature over 38 °C) from the 3rd dpi, lasting for a maximum of 3 days (Fig. 2a). Erythrocytes, leukocytes and platelets counts are shown in Fig. 2b–d.

### IFA

**Fig. 3:**
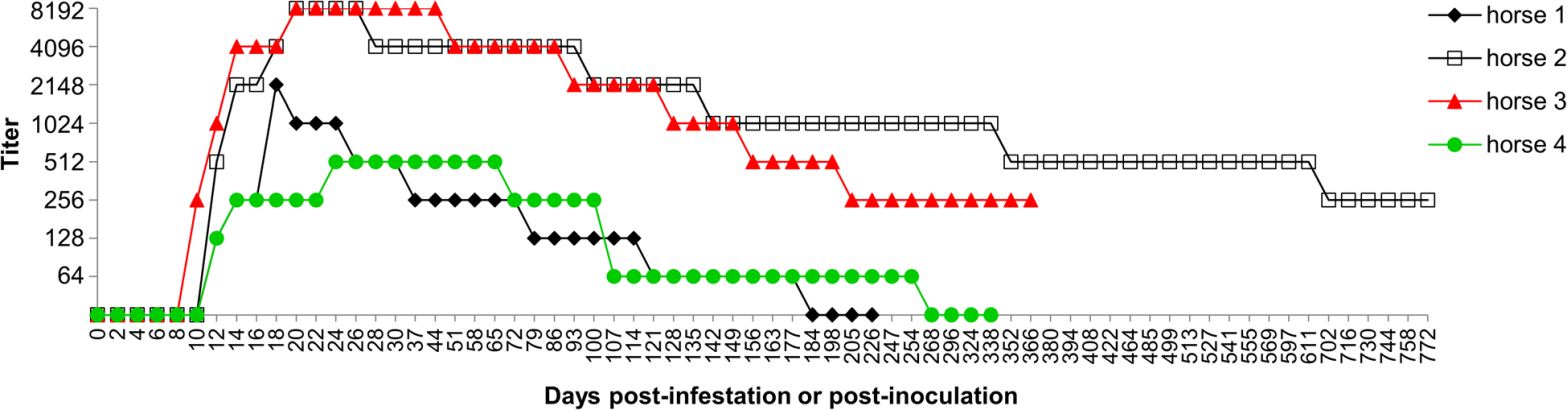
Antibody titers (IFA ≥ 64) in horses experimentally infected with *Rickettsia rickettsii. Abbreviation*: IFA, indirect immunofluorescence assay

Anti-*R. rickettsii* antibodies (titers ≥ 64) were first detected at 10 dpi (horse 3) or 12 dpi (horses 1, 2 and 4). Endpoint titers peaked at 18 to 24 dpi and entered the decreasing curve between 20 and 72 dpi. The highest titers were 512 for horse 4, 2,048 for horse 1, and 8,192 for horses 2 and 3. Horses 1 and 4 remained seropositive until 177 dpi and 254 dpi, respectively. The maximum period of antibody persistence could not be determined for horses 2 and 3 because the study was finished before they became seronegative. Horse 2 was monitored until 772 dpi, when the titer was 256. Horse 3 was monitored up to 366 dpi, when the titer was 256 (Fig. 3).

Comparative tests between antigens showed that all *R. rickettsii-*positive serum samples were also positive for *R. parkeri*, while some of these samples were also positive for the remaining four antigens. Titers for *R. rickettsii* were higher or equal to those for other antigens. On two occasions (on the days of peak titer in horses 1 and 3), the titer for *R. rickettsii* was at least 4-fold higher than the titers for the other five *Rickettsia* species (Table 1).

**Table 1:** IFA end-point titers for six *Rickettsia* species in horses experimentally infected with *Rickettsia rickettsii*

Horse	Days post-infection	IFA titers for *Rickettsia* antigens					
		*R. rickettsii*	*R. parkeri*	*R. amblyommii*	*R. rhipicephali*	*R. bellii*	*R. felis*
1	0	–	–	–	–	–	–
1	12	128	128	64	–	64	–
1	18	2,048	256	256	256	256	128
1	86	128	128	64	64	64	–
1	177	64	64	–	–	–	–
2	0	–	–	–	–	–	–
2	12	512	256	256	128	256	–
2	20	8,192	4,096	2,048	2,048	4,096	1,024
2	611	512	256	–	–	–	–
2	772	256	128	–	–	–	–
3	0	–	–	–	–	–	–
3	10	256	256	64	64	64	–
3	20	8,192	2,048	1,024	2,048	512	2,048
3	366	256	128	128	128	–	128
4	0	–	–	–	–	–	–
4	12	128	64	–	–	–	–
4	24	512	256	128	128	128	256
4	100	256	128	64	–	–	–
4	254	64	64	64	64	64	64

*Abbreviations*: IFA, indirect immunofluorescence assay–, nonreactive at titer ≥ 64

### Search for *Rickettsia* in horse blood

All blood samples from the four horses were negative by real-time PCR throughout the evaluating period. No guinea pigs inoculated with horse blood showed hyperthermia or clinical signs compatible with *R. rickettsii* infection. These guinea pigs were seronegative for *R. rickettsii* at 21 dpi.

### Tick acquisition and transmission of rickettsiae

Among the unfed nymphs and adults that had fed on horse 1 as larvae and nymphs, respectively, 2/90 (2.2 %) nymphs and 2/56 (3.6 %) adults revealed rickettsial DNA by real-time PCR (Table 2).

**Table 2:** Real-time PCR on ticks that were exposed to rickettsiae by feeding on *Rickettsia rickettsii-*infected horses

Group	Horse	No. of positive ticks in real-time PCR/no. of tested ticks					Total
		Unfed nymphs (fed as larvae on horses and molted to nymphs)	Unfed adults (fed as larvae on horses, fed as nymphs on rabbits, and molted to adults)	Unfed adults (fed as nymphs on horses and molted to adults)	Fed adults (fed as nymphs on horses and fed as adults on rabbits)^a^	Fed adults (fed as adults on horses)^a^	
Group 1^b^	1	2/90 (2.2 %)		2/56 (3.6 %)	0/83 (0)	12/51 (2.4 %)	16/280 (5.7 %)
	2				0/70 (0)	0/29 (0)	0/99 (0)
Group 2^b^	3		0/7 (0)		1/189 (0.5 %)	0/21 (0)	1/217 (0.5 %)
	4		1/178 (0.6 %)		0/69 (0)	0/22 (0)	1/269 (0.4 %)
Total		2/90 (2.2 %)	1/185 (0.5 %)	2/56 (3.6 %)	1/411 (0.2 %)	12/123 (9.8 %)	18/865 (2.1 %)

^a^Engorged females collected from horses or rabbits were tested by real-time PCR after oviposition. Males recovered from horses or rabbits were tested soon after collection

^b^Rickettsial infection in Group 1 (horses 1 and 2) were via *Rickettsia rickettsii*-infected *Amblyomma sculptum* ticks, whereas Group 2 (horses 3 and 4) were inoculated intraperitoneally with macerated organs from *R. rickettsii*-infected guinea pigs

Horse-derived ticks that successfully fed on rabbits consisted of 194 nymphs and 86 adults from horse 1; 77 adults from horse 2; 7 nymphs and 205 adults from horse 3; and 191 nymphs and 73 adults from horse 4. None of the infested rabbits seroconverted to *R. rickettsii* or presented any clinical signs compatible with rickettsiosis.

Considering all acquisition tested ticks, including those fed only on horses and those fed sequentially on horses and rabbits, 2.1 % (18/865) were positive by real-time PCR (Table 2). The real-time PCR positivity among ticks that had fed on horses as larvae, nymphs, or adults were 1.1 % (3/275), 0.6 % (3/467) and 9.8 % (12/123), respectively. However, when these ticks were subjected to *ompA* gene-based conventional PCR, just one was positive and its sequencing was faulty due to low DNA concentration. This tick was a nymph that fed as larva on horse 1, and was not fed on rabbit later, so it was not possible to check the transmission capability of this individual tick.

### Infectivity test of ticks and inocula

The rabbits that were infested simultaneously to Group 1 horses with *R. rickettsii-*infected ticks, and the guinea pigs that were inoculated with *R. rickettsii* simultaneously to Group 2 horses showed apathy, fever and necrosis of ears and scrotum. One rabbit and two guinea pigs died and their organs tested positive in real-time PCR. All surviving animals were seropositive at 21 dpi. All ticks that fed on rabbits were positive by real-time PCR, as were 100 % (27/27) and 97 % (34/35) of the transmission-fed ticks that fed on horses 1 and 2, respectively. From six ticks selected for rickettsial isolation, *R. rickettsii* was successfully isolated from five of them in cell culture, guinea pig inoculation, or both.

## Discussion

In this study, *R. rickettsii-*infected horses showed no marked clinical changes and the highest rectal temperature observed was 38.4 °C. Rickettsemia was not detected by real-time PCR on horse blood or by guinea pig bioassay. Detection of rickettsia in blood is usually difficult because this bacterium multiplies within endothelial cells, and a low number of these cells are found free in the circulation in cases of mild illness [33]. However, the inoculation of guinea pigs is a sensitive method, as they are very susceptible to *R. rickettsii*, even when receiving low infectious doses [13].

Ricketts [18] and Heinemann & Moore [19] observed temperatures above 39 °C lasting for a maximum of 4 days in horses inoculated with USA strains of *R. rickettsii*. In these animals, blood was infective for guinea pigs only at the peak fever day (i.e. the temperature seems to follow the level of bacteremia). Although tick acquisition feeding was not tested in those earlier works, the very short bacteremic period suggests few ticks would acquire the infection. Discrepancies in relation to the occurrence of fever and bacteremia between studies could be attributed to possible differences in virulence of strains and infectious dose.

Among the acquisition feeding ticks that fed on horses, only 2.1 % were positive for *Rickettsia* through real-time PCR, and among these, only one was positive in conventional PCR targeting the *ompA* gene. As the real-time PCR is more sensitive than the conventional PCR, this result could be attributed to low bacterium load in ticks, which is corroborated by the high threshold cycle values (more than 31) presented by the positive tick samples (data not shown). The absence of illness or seroconversion in rabbits infested with these ticks suggest the rickettsial infection has not been successfully established in ticks, the bacterium did not multiply effectively, and possibly failed to reach the salivary gland. This finding indicates that the horses did not develop a level of rickettsemia sufficient to infect ticks effectively, so these arthropods were not able to transmit the bacterium to the next host.

Another aspect that indeed reduces the involvement of horses in the transmission cycle of *R. rickettsii* is the low prolificacy of this vertebrate species, which has a long pregnancy period and usually produces only one foal per pregnancy [34]. As vertebrate hosts develop transient bacteremia and after this period acquire long-lasting immunity, a condition for considering an animal efficient amplifier host is that it gives birth to numerous offspring in a short time, resulting in a continual replacement of susceptible individuals in a particular region [1]. Regarding horses, the only way of abundant replacement would be frequent introduction of individuals from other locations.

All horses developed humoral immune response, which has been reported in several studies that found seropositive horses under natural conditions [15–17]. Earlier studies have already shown indirect evidence of antibody production in *R. rickettsii*-inoculated horses by showing the preventive and curative effect of equine hyperimmune serum on guinea pigs [19, 35]. However, the use of equine serum in human patients would be limited because the protective effect was observed only when the serum was administered during the first 3 days of infection (before the onset of symptoms) and hypersensitivity reactions might occur [19, 35]. Due to their notable humoral response, horses can be used as efficient sentinels for detecting the circulation of *R. rickettsii* in areas where the primary vector uses horses as hosts, as is the case of *A. sculptum*. The IgG antibody persistence was approximately 6, 26, 12 and 8 months for horses 1, 2, 3 and 4, respectively. Monitoring of horses 2 and 3 was ceased before they became seronegative, i.e. the antibodies probably lasted longer. Other studies followed the animals up to 6 months (dogs and opossums) [36], 10 months (cotton rat *Sigmodon hispidus*) [37], or 11 months (desert woodrat *Neotoma lepida*) [38], when the antibodies were still present. Because of the prolonged antibody persistence, the selection of horses to be used as sentinels should preferably include those animals that were born in the target area, or that were living there for over 2 years.

Cross-reactions among *R. rickettsii* and the other five *Rickettsia* antigens were observed, although no serum elicited a heterologous endpoint titer higher than the homologous titer to *R. rickettsii*. Some studies used the finding of a titer for a specific rickettsia at least 4-fold higher than the titer for any other antigens as criterion to consider this rickettsia, or closely related genotype, the probable responsible for animal infection (homologous antigen) [16, 36]. Only two sera, corresponding to the peak titer days of two horses, obeyed this criterion. Thus, the determination of homologous antigen in naturally infected horses would probably occur on few occasions and more often when the endpoint titer is high, making other techniques such as cross-absorption [16] necessary to detect which *Rickettsia* species is involved in the infection.

The infective dose inoculated into horses could not be quantified, although infectivity tests of infected ticks and guinea pig inocula showed both contained viable bacteria pathogenic to rabbits and guinea pigs. The transmission adult ticks that fed on horses 1 and 2 had infection rates of 100 % and 97 %, respectively. Considering that each horse from group 1 was infested with 60 ticks that remained in the animals for up to 24 days, it can be inferred that the horses received considerable amounts of bacteria via tick feeding. These results validated our results, i.e. horses did not develop clinical illness due to *R. rickettsii* and did not serve as amplifier hosts of *R. rickettsii* for *A. sculptum* ticks. Finally, our results are also validated by three previous studies that used exactly the same protocols in our laboratory (including the same tick and rickettsial strains), and showed that guinea pigs, capybaras and opossums developed rickettsemia and served as infection sources for *A. sculptum* ticks [8, 12, 13].

The strain of *R. rickettsii* used in this study was isolated from *A. aureolatum* tick, not from *A. sculptum*. This strain was used because when the study was initiated there was no Brazilian strain isolated from *A. sculptum* ticks available, which was obtained later [39]. However, the absence of BSF cases in some areas of Brazil where horses are alone the main hosts of *A. cajennense* (*s.l*.) [1], coupled with the low prolificacy of horses, suggest that another strain would not change the results and reinforce the idea that horses are not amplifier hosts of *R. rickettsii*.

Further studies are needed to evaluate other *R. rickettsii* strains adapted to different populations of *A. cajennense* (*s.l*.), the role of co-feeding on non-bacteremic hosts in acquisition of *R. rickettsii* by ticks, and the mechanism of immunity against rickettsiae in horses.

### Conclusions

Our results indicate that horses do not have competence to transmit *R. rickettsii* to *A. sculptum* ticks, and that probably they are not relevant in the maintenance cycle of this bacterium in nature. Moreover, *R. rickettsii*-infected horses did not show illness, but present detectable IgG antibodies with high titers and long-lasting persistence.

## Abbreviations

BHI: Brain heart infusion
BSF: Brazilian spotted fever
dNTP: Deoxynucleotide triphosphate
dpi: Days post-inoculation or post-infestation
EDTA: Ethylenediaminetetraacetic acid
IFA: Indirect immunofluorescence assay
